# Sampling collections and metadata of planorbidae (Mollusca: Gastropoda) in Brazil: a comprehensive analysis of the Oswaldo Cruz Institute’s Mollusk Collection from 1948 to 2023

**DOI:** 10.46471/gigabyte.102

**Published:** 2023-12-07

**Authors:** Silvana Carvalho Thiengo, Mariana Gomes Lima, Alexandre Bonfim Pinheiro da Silva, Raiany Thuler Nogueira, Flávia Cristina dos Santos Rangel, Suzete Rodrigues Gomes

**Affiliations:** ^1^ Laboratório de Referência Nacional para Esquistossomose – Malacologia, Instituto Oswaldo Cruz, Fundação Oswaldo Cruz (LRNEM/IOC/Fiocruz), Brasil

## Abstract

Planorbidae comprises approximately 40 genera of freshwater gastropods, including roughly 250 species. Among the Planorbidae subfamilies, the significance of Planorbinae is due to its genus *Biomphalaria*, whose species are intermediate hosts of the trematode *Schistosoma mansoni* Sambon, 1907, which causes schistosomiasis in humans and animals. Here, we present the analysis of the dataset of Planorbidae housed in the Collection of Mollusks of the Oswaldo Cruz Institute, with a special focus on *Biomphalaria* species. This dataset includes 7,267 lots originating from 55 countries, representing 20 genera and 75 species collected from 1948 to 2023. Collections were performed in all regions of Brazil, comprising specimens from 26 states and the Federal District, particularly from the Southeast and Northeast. Within the dataset, *Biomphalaria* includes 3,926 lots of 31 species from 42 countries. These records will help improve our comprehension of schistosomiasis transmission dynamics and the geographic distributions of these medically important species.

## Data description

### Background and context

**Figure 1.**
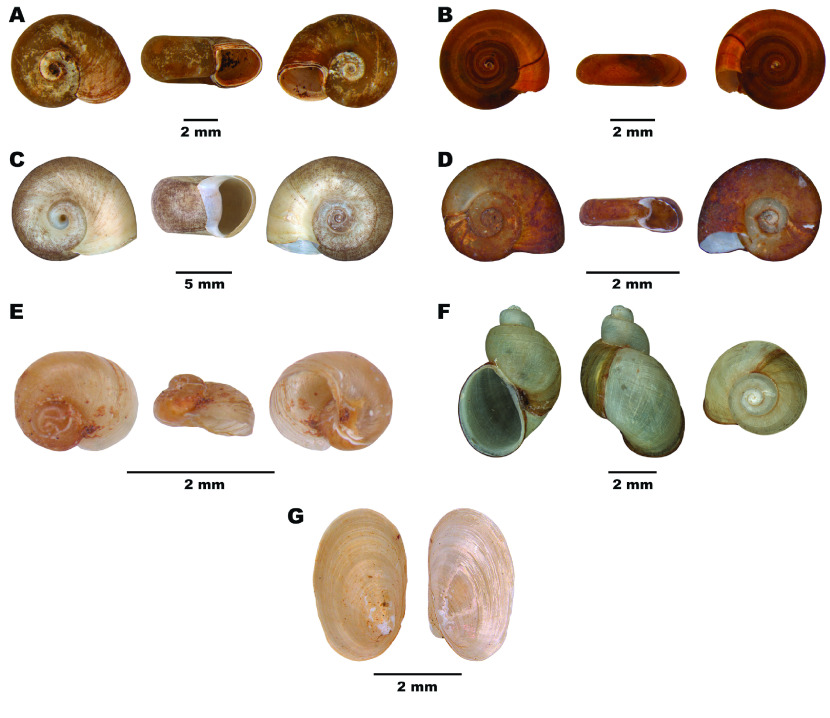
Diversity of shell forms in Planorbidae from Brazil. (A) *Biomphalaria straminea* (CMIOC 5612), (B) *Drepanotrema lucidum* (CMIOC 5573), (C) *Helisoma duryi* (CMIOC 2318), (D) *Antillorbis nordestensis* (CMIOC 4550), (E) *Acrorbis petricola* (CMIOC 2744), (F) *Plesiophysa dolichomastix* (CMIOC 2041), and (G) *Gundlachia ticaga* (CMIOC 14689).

The family Planorbidae includes around 40 genera of freshwater gastropods, with around 250 species widely distributed [1]. In this family, the subfamily Planorbinae includes six genera reported in Brazil: *Acrorbis*, *Antillorbis*, *Biomphalaria*, *Drepanotrema*, *Helisoma*, and *Plesiophysa* [2] (Figure 1). *Biomphalaria* includes species that act as intermediate hosts of the trematode *Schistosoma mansoni*, which causes schistosomiasis mansoni [3]. In Brazil, *S. mansoni* utilizes three species of the genus *Biomphalaria* as its natural intermediate hosts: *Biomphalaria glabrata* (Say, 1818), *B. straminea* (Dunker, 1848), and *Biomphalaria tenagophila* (d’Orbigny, 1835) [4, 5].

 In this paper, we contributed a dataset derived from the Planorbidae species deposited in the Oswaldo Cruz Institute’s Mollusk Collection, mainly from Brazil, but also from numerous other countries. This material was mainly the result of decades of study in freshwater ecosystems by Dr. Wladimir Lobato Paraense, known for his studies on the biology and taxonomy of Brazilian planorbids, and by his team in the laboratory of Malacology of the Oswaldo Cruz Institute. Many other specialists also contributed and examined several specimens from this collection. In 1948, when Dr. Wladimir Lobato Paraense worked at the Public Health Special Service, a Brazilian institution responsible for the control of parasitic diseases in the Rio Doce Valley in Minas Gerais, including schistosomiasis [6, 7], he began his studies on the mollusks involved in the transmission of schistosomiasis in Brazil, creating the collection.

Currently, this collection includes mainly freshwater and land gastropods involved in the transmission of other parasitic diseases, such as fascioliasis and angiostrongyliasis, both cerebral and abdominal, but also includes gastropod species that cause economic losses in agriculture (mainly exotic species) and among native species from the Brazilian Biomes. The collection contributes to science, research, and education. It also serves as a repository of knowledge about Brazilian and global mollusk biodiversity [6].

The datasets presented in this study consist of metadata associated with each batch of Planorbidae specimens, featuring varying numbers of specimens. We filled in the obligatory fields and have successfully passed screening using the integrated publishing toolkit (IPT) of the Fundação Oswaldo Cruz (FIOCRUZ). For each lot of Planorbidae, our dataset includes fields providing, in Darwin Core Standard format, the following information: (i) taxonomy (kingdom, phylum, class, order, family, genus, specificEpithet, verbatimIdentification, infraspecificEpithet, scientificName, scientificNameAuthorship, taxonRank); (ii) collection details, including the collectors, collection date, collection site description (verbatimEventDate, eventTime, habitat, samplingProtocol); (iii) geolocation data (stateProvince, county, locality, locationRemarks, verbatimLatitude, verbatimLongitude, decimalLatitude, decimalLongitude, geodeticDatum); and (iv) catalog reference data (otherCatalogNumbers). This dataset is also available in the Sistema de Informação sobre a Biodiversidade Brasileira (SiBBr; i.e., Information System on the Brazilian Biodiversity), which integrates data and information, constituting the Brazilian Node of the Global Biodiversity Information Facility (GBIF), in an online platform for public use [8].

## Methods

**Figure 2.**
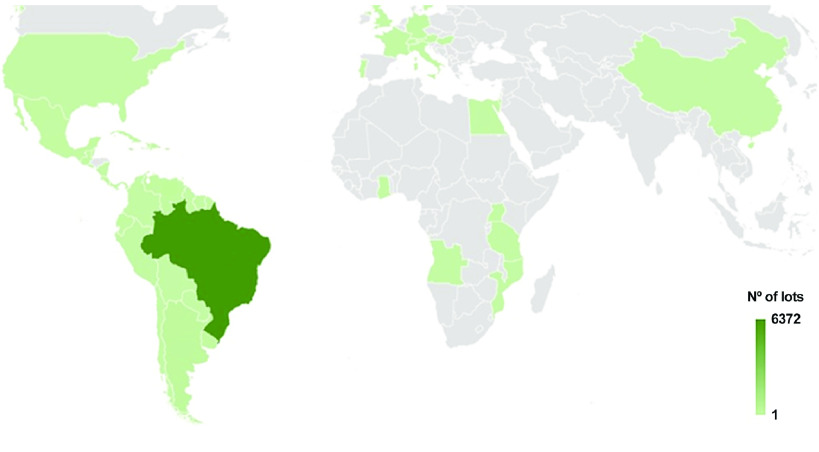
Spatial coverage of the occurrence dataset of Planorbidae, from 1948 to 2023, based on the CMIOC, including mainly lots are from Brazil (6,372 lots).

This study included all reports of Planorbidae genera and species from the dataset obtained from the Oswaldo Cruz Institute’s Mollusk Collection (CMIOC). These lots are mainly from Brazil, but also include material from more than 50 countries and continents (Figure 2). The temporal coverage of the Planorbidae dataset is from 1948 to 2023.

 All mollusks were morphologically identified in the laboratory to their genus and species based on shell and anatomical characteristics through specimen dissections, literature, and comparison with the lots deposited in the CMIOC [2, 9–12].

## Data validation and quality control

Over the years, Dr. Lobato Paraense and his group published several works on planorbids from Brazil [9, 13–33]. Their works described and redescribed species of Planorbinae, reinforcing the importance of planorbids in transmitting diseases. Data validation was also done via the GBIF data-validator tool upon data submission [8].

## Results

The CMIOC has records of representatives of the family Planorbidae (Table 1) from 55 out of the 193 countries recognized by the United Nations in 2023, spanning across four out of the six continents. These countries include Germany, Angola, Antigua, Antigua and Barbuda, Argentina, Austria, Barbados, Belize, Bolivia, Brazil, Chile, China, Costa Rica, Colombia, Cuba, Egypt, El Salvador, Ecuador, United States of America, France, Ghana, Guadeloupe, Guatemala, Guyana, French Guiana, Haiti, Hong Kong, Hungary, England, Israel, Italy, Jamaica, Martinique, Mexico, Mozambique, Nicaragua, Panama, Paraguay, Peru, Puerto Rico, Portugal, Dominican Republic, Saint Croix, Saint Thomas, Saint Vincent, Saint Lucia, Sweden, Suriname, Tahiti, Tanzania, Trinidad, Uganda, Uruguay, and Venezuela. These reports cover the following coordinates: 90°0′0′′S and 90°0′0′′N Latitude; 180°0′0′′W and 180°0′0′′E Longitude.

**Table 1:** Genera and species of the Planorbidae family deposited in the CMIOC.

Genus	Species
*Acrobis*	*Acrorbis petricola*
*Antillorbis*	*Antillorbis nordestensis, Antillorbis salleanus*
*Biomphalaria*	*Biomphalaria adowensis, Biomphalaria alexandrina, Biomphalaria amazonica, Biomphalaria andecola, Biomphalaria choanomphala, Biomphalaria costata, Biomphalaria cousini, Biomphalaria glabrata, Biomphalaria havanensis, Biomphalaria helophila, Biomphalaria intermedia, Biomphalaria kuhniana, Biomphalaria nicaraguana, Biomphalaria obstructa, Biomphalaria occidentalis, Biomphalaria oligoza, Biomphalaria orbignyi, Biomphalaria pallida, Biomphalaria peregrina, Biomphalaria pfeifferi, Biomphalaria prona, Biomphalaria pucaraensis, Biomphalaria schrammi, Biomphalaria sericea, Biomphalaria stanleyi, Biomphalaria straminea, Biomphalaria subprona, Biomphalaria sudanica, Biomphalaria tenagophila, Biomphalaria thermala, Biomphalaria trigyra.*
*Drepanotrema*	*Drepanotema anatinum, Drepanotrema beltrani, Drepanotrema cimex, Drepanotrema depressissimum, Drepanotrema heloicum, Drepanotrema kermatoides, Drepanotrema limayanum, Drepanotrema lucidum, Drepanotrema pfeifferi, Drepanotrema pileatum, Drepanotrema simonsi, Drepanotrema surinamense*
*Ferrissia*	*Ferrissia fragilis*
*Gundlachia*	*Gundlachia radiata, Gundlachia ticaga*
*Gyraulus*	*Gyraulus acronicus, Gyraulus albus, Gyraulus boetzkesi, Gyraulus crista, Gyraulus percarinatus*
*Hebetancylus*	*Hebetancylus moricandi*
*Helisoma*	*Helisoma anceps, Helisoma campanulatum, Helisoma caribaeum, Helisoma corpulentum, Helisoma duryi, Helisoma foveale, Helisoma peruvianum, Helisoma trivolvis*
*Hippeutis*	*Hippeutis complanatus*
*Laevapex*	*Laevapex diaphanous*
*Planorbarius*	*Planorbarius corneus*
*Planorbis*	*Planorbis boissyi, Planorbis canonicus, Planorbis corneus, Planorbis metidjensis, Planorbis planorbis, Planorbis salleanus*
*Segmentina*	*Segmentina nitida*
*Tropicorbis*	*Tropicorbis riisei*
*Uncancylus*	*Uncancylus concentricus*

In Brazil, the geographical distribution covers all five territorial regions, spanning the 26 states and the Federal District. The present database presents records of the occurrence of representatives of the family Planorbidae in a total of 592 municipalities (Midwest: recorded in 32 municipalities; Northeast: 120 municipalities; North: 27 municipalities; Southeast: 339 municipalities; and South: 74 municipalities). *Biomphalaria* is the most well-represented genus in CMIOC, including lots also from Africa, Asia, and Latin and North Americas, with a total of 3,926 lots. These specimens are registered in the following countries: Angola, Antigua, Argentina, Barbados, Belize, Bolivia, Chile, China, Costa Rica, Cuba, Egypt, El Salvador, Ecuador, United States, Ghana, Guadeloupe, Guatemala, Guyana, French Guiana, Haiti, Hong Kong, Jamaica, Martinique, Mexico, Mozambique, Nicaragua, Panama, Paraguay, Peru, Puerto Rico, Dominican Republic, Santa Lucia, Suriname, Tanzania, Trinidad, Uganda, Uruguay, and Venezuela (Table 2). Another well-represented genus of Planorbinae is *Drepanotrema*, which includes twelve species from different countries, with 2,312 lots (Table 3).

**Table 2:** Origin of the lots of *Biomphalaria* deposited in the CMIOC.

Country	Species	Country	Species	Country	Species	Country	Species
Angola	*B. adowensis*	Ecuador	*B. cousini*	Hong Kong	*B. straminea*	Dominican Republic	*B. glabrata*
Antigua	*B. glabrata*		*B. peregrina*	Jamaica	*B. helophila*		*B. helophila*
Argentina	*B. intermedia*		*B. sericea*		*B. pallida*		*B. straminea*
	*B. oligoza*		*B. trigyra*	Martinique	*B. glabrata*	Saint Lucia	*B. glabrata*
	*B. orbignyi*	United States of America	*B. glabrata*		*B. kuhniana*	Suriname	*B. glabrata*
	*B. peregrina*		*B. havanensis*		*B. straminea*		*B. kuhniana*
	*B. straminea*		*B. peregrina*	Mexico	*B. obstructa*		*B. straminea*
	*B. tenagophila*		*B. obstructa*	Mozambique	*B. pfeifferi*	Tanzania	*B. choanomphala*
Barbados	*B. helophila*	Ghana	*B. pfeifferi*	Nicaragua	*B. helophila*		*B. pfeifferi*
Belize	*B. helophila*	Guadeloupe	*B. glabrata*		*B. nicaraguana*		*B. sudanica*
	*B. obstructa*		*B. kuhniana*		*B. obstructa*	Trinidad	*B. straminea*
Bolivia	*B. andecola*		*B. schrammi*	Panama	*B. kuhniana*	Uganda	*B. stanleyi*
	*B. pucaraensis*	Guatemala	*B. helophila*	Paraguay	*B. peregrina*	Uruguay	*B. straminea*
Chile	*B. costata*		*B. obstructa*		*B. occidentalis*		*B. tenagophila*
	*B. peregrina*		*B. subprona*		*B. tenagophila*		*B. tenagophila guaibensis*
	*B. thermala*	Guyana	*B. glabrata*		*B. straminea*	Venezuela	*B. glabrata*
China	*B. straminea*		*B. schrammi*	Peru	*B. andecola*		*B. peregrina*
Costa Rica	*B. helophila*		*B. straminea*		*B. helophila*		*B. prona*
	*B. straminea*	French Guiana	*B. glabrata*		*B. peregrina*		*B. straminea*
Cuba	*B. havanensis*	Haiti	*B. glabrata*		*B. pucaraensis*		
	*B. helophila*		*B. havanensis*		*B. tenagophila*		
Egypt	*B. alexandrina*		*B. helophila*		*B. trigyra*		
El Salvador	*B. helophila*		*B. pallida*	Puerto Rico	*B. glabrata*		
	*B. obstructa*		*B. obstructa*		*B. helophila*		
			*B. straminea*		*B. peregrina*		

**Table 3:** Origin of the lots of *Drepanotrema* deposited in the CMIOC.

Species	Country	Number of lots	Species	Country	Number of lots
*Drepanotrema anatinum*	Argentina	11	*Drepanotrema kermatoides*	Argentina	22
	Belize	1		Brazil	32
	Brazil	521		Ecuador	2
	Costa Rica	1		Paraguay	1
	Ecuador	1		Peru	10
	Guatemala	3		Uruguay	12
	Guyana	1	*Drepanotrema limayanum*	Peru	6
	Haiti	2	*Drepanotrema pfeifferi*	Argentina	1
	Jamaica	4		Chile	3
	Mexico	1	*Drepanotrema pileatum*	Brazil	8
	Nicaragua	1	*Drepanotrema lucidum*	Antigua	1
	Panama	1		Argentina	23
	Puerto Rico	5		Barbados	1
	Dominican Republic	2		Belize	2
	Saint Lucia	1		Brazil	808
	Suriname	2		Ecuador	2
	Trinidad	3		United States	1
	Uruguay	1		Guadeloupe	4
	Uruguay	2		Guatemala	1
	Venezuela	2		Haiti	1
*Drepanotrema beltrani*	Mexico	1		Jamaica	8
*Drepanotrema cimex*	Argentina	7		Mexico	3
	Brazil	369		Nicaragua	2
	Haiti	1		Paraguay	5
	Jamaica	3		Puerto Rico	6
	Puerto Rico	1		Dominican Republic	2
	Uruguay	3		Saint Vincent	1
	Venezuela	3		Saint Lucia	3
*Drepanotrema depressissimum*	Antigua	1		Uruguay	4
	Argentina	13		Trinidad	2
	Barbados	2		Venezuela	1
	Brazil	295	*Drepanotrema simonsi*	Puerto Rico	1
	Costa Rica	1	*Drepanotrema surinamense*	Costa Rica	2
	Guadeloupe	7		Ecuador	2
	Nicaragua	2		Guadeloupe	1
	Paraguay	1		Guyana	1
	Peru	1		Panama	4
	Saint Lucia	2		Suriname	4
	Uruguay	3			
	Venezuela	1			
*Drepanotrema heloicum*	Argentina	15			
	Brazil	3			
	Uruguay	8			

Considering lots from Brazil, the collection at CMIOC includes representatives from all 11 species of *Biomphalaria* that occur nationwide. Among them, three species play a significant role in the biological cycle of Brazil [33] and have a wide distribution. Together, these species account almost 40% of the total Planorbidae lots in the collection. Specifically, *B. straminea* is the most represented species with 1,257 lots, while *B. tenagophila*, and *B. glabrata* account for 811 and 654 lots, respectively.

**Figure 3.**
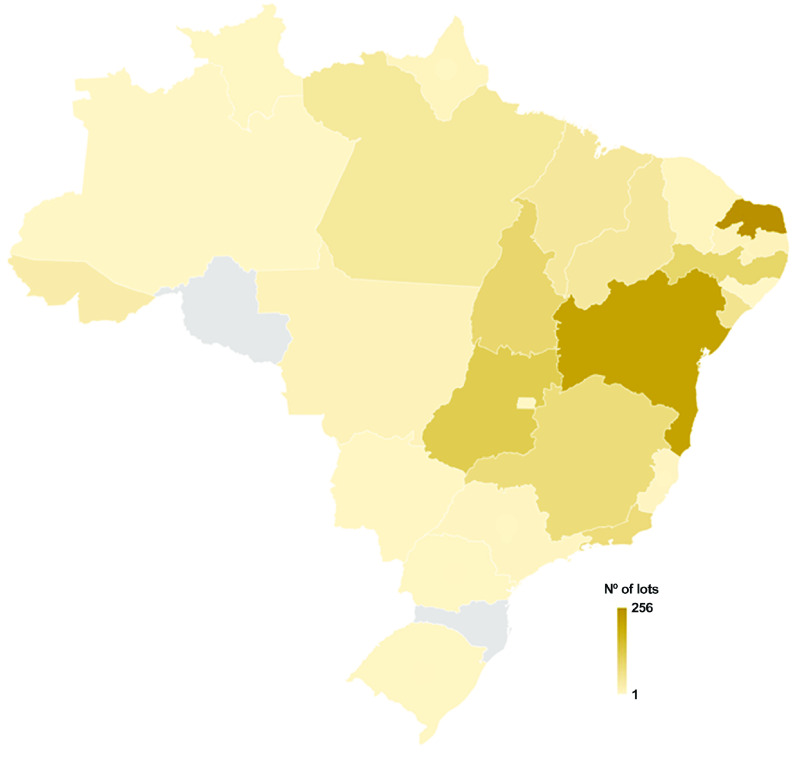
Spatial coverage of the occurrence dataset of *B. straminea*, from 1948 to 2023, based on the CMIOC, showing that most lots are from Rio Grande do Norte, with 256 lots.

**Figure 4.**
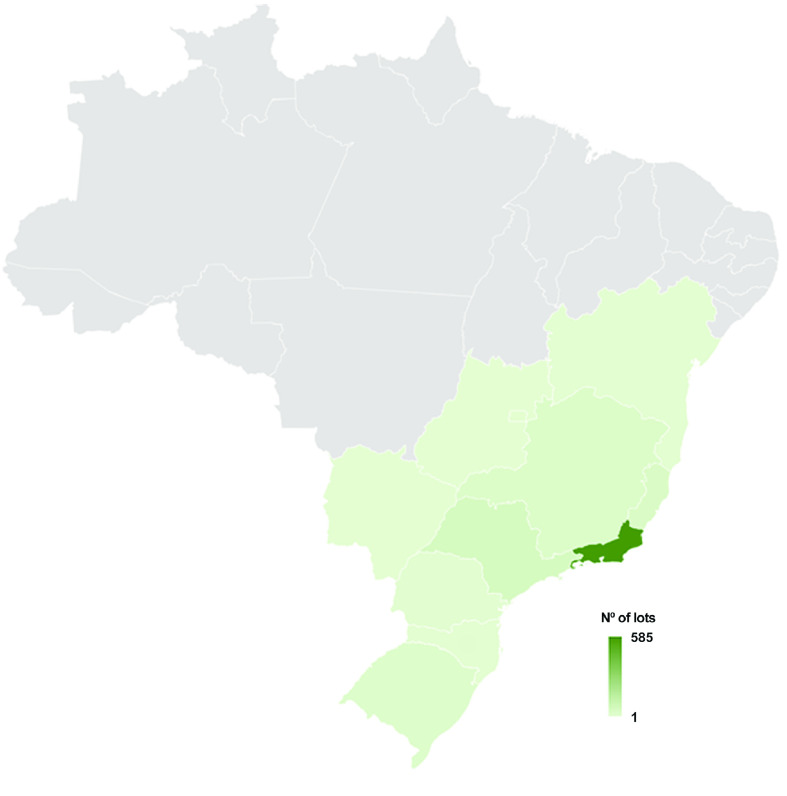
Spatial coverage of the occurrence dataset of *B. tenagophila*, from 1948 to 2023, based on the CMIOC, showing that most lots are from Rio de Janeiro State, with a total of 585 lots. The second more represented State is São Paulo, with 54 lots.

**Figure 5.**
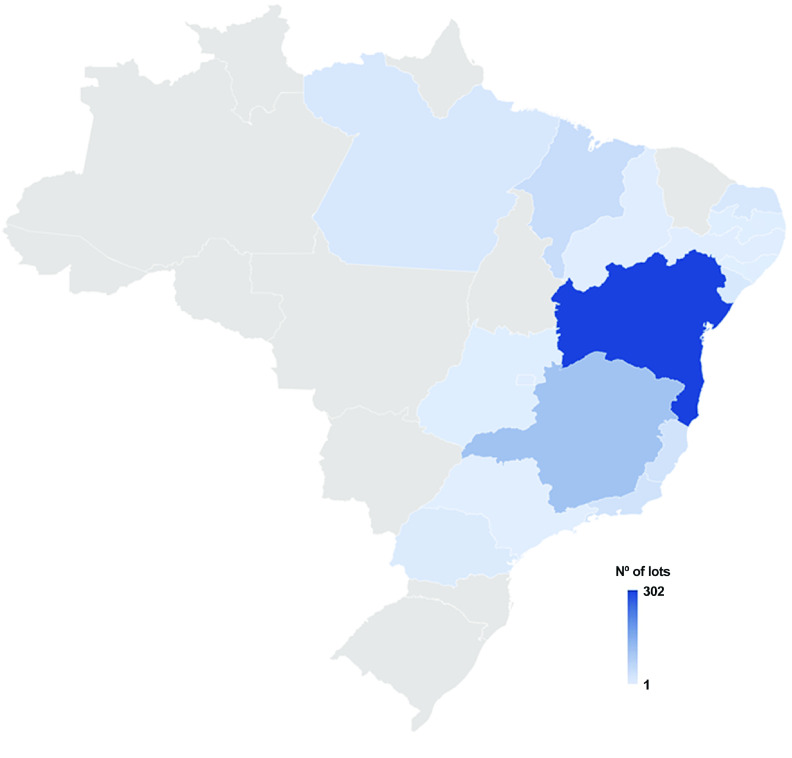
Spatial coverage of the occurrence dataset of *B. glabrata*, from 1948 to 2023, based on the CMIOC, showing that most lots are from Bahia, with 302 lots.

The three species of *Biomphalaria* found in Brazil and recorded at CMIOC exhibit distinct geographic distributions within the country. However, there are certain states where these species overlap, indicating areas of coexistence (Figures 3–5).

*B. straminea* has the broadest geographic range among the three species in Brazil. It can be found in various states, from the North to the South of the country. Its distribution encompasses regions such as the Amazon, the Cerrado, the Caatinga, and the Atlantic Forest. The extensive presence of *B. straminea* highlights its significance as an intermediate host for the parasitic trematode. For a complete record of *B. straminea* in Brazil [34], CMIOC only lacks samples from the states of Santa Catarina and Roraima (Figure 3). On the other hand, *B. tenagophila* has a more restricted distribution compared to *B. straminea*. *B. tenagophila* is commonly found in areas of the South and Southeast of Brazil, primarily encompassing the states of São Paulo, Paraná, Santa Catarina, and Rio Grande do Sul (Figure 4). Finally, *B. glabrata* has a less limited geographic distribution than *B. tenagophila* in Brazil. Specifically, *B. glabrata* is predominantly found in coastal areas, particularly in the Northeast region of the country (Figure 5).

## Data validation and quality control

Planorbidae specimens were identified by experienced taxonomists. The dataset is in Darwin Core format, and all mandatory fields are present and have undergone screening in the FIOCRUZ IPT.

## Reuse potential

**Figure 6.**
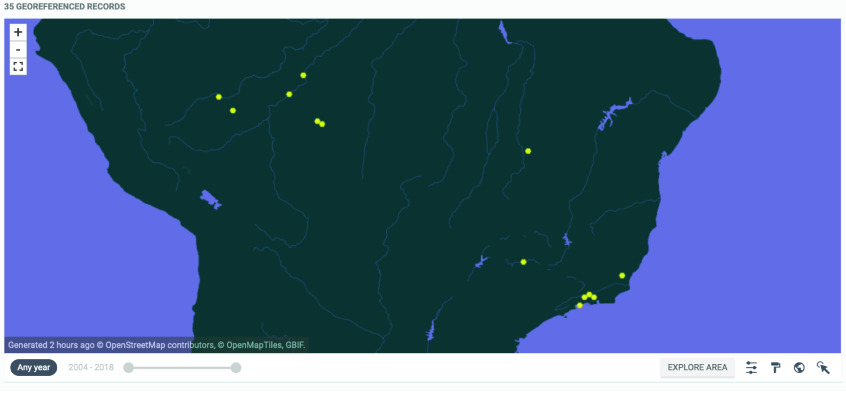
Interactive map of the georeferenced occurrences hosted by GBIF [8]. https://www.gbif.org/dataset/2bd86564-44c0-4317-a04e-79b544a84a06

The presented dataset is important because it provides information on the distribution of Planorbidae, Planorbinae, and *Biomphalaria* in Brazil based on a renowned collection of medical malacology (i.e., CMIOC), traditionally known for its studies within the country. This dataset can provide the basis for future studies in evolution, ecology, and epidemiology, among others, especially for species of medical interest from the public health perspective. An important point of the current collection is the first recording of *B. straminea* in Amapá, serving as an important reference for research into the biodiversity of Planorbinae. These data expand the distribution of these species and provide occurrence information on the other species of this genus in Brazil. In addition to supporting the surveillance and control of schistosomiasis in Brazil, these data also contribute to the knowledge of *Biomphalaria* biodiversity. They are also an important resource for managing the CMIOC (Figure 6).

## Data Availability

The dataset used in this article is published through the FIOCRUZ – Oswaldo Cruz Foundation IPT – and is provided under a CC0 waiver from GBIF [8] and in the SiBBr repository [34].

## Editor’s note

This paper is part of a series of Data Release articles working with GBIF and supported by TDR, the Special Programme for Research and Training in Tropical Diseases hosted at the World Health Organization, in order to publish datasets on vectors of human diseases [35].

## Abbreviations

CMIOC, Collection of Mollusks of the Oswaldo Cruz Institute; FIOCRUZ, Fundação Oswaldo Cruz; GBIF, Global Biodiversity Information Facility; IPT: integrated publishing toolkit; SiBBr, Sistema de Informação sobre a Biodiversidade Brasileira.

## References

[1] Strong EE, Gargominy O, Ponder WF Global diversity of gastropods (Gastropoda; Mollusca) in freshwater. Freshwater Animal Diversity Assessment, 2008; 595: 149–166. doi:10.1007/s10750-007-9012-6.

[2] Fernandez MA, Feitosa ES, Thiengo SC. Planorboidea, Planorbidae, Planorbinae. In: Damborenea C, Rogers DC, Thorph HJ (eds), Key to Neotropical and Antarctic Fauna. vol. 5, (4): Academic Press, 2020; pp. 313–327.

[3] World Health Organization. Schistosomiasis. 2020. Retrieved from https://www.who.int/news-room/fact-sheets/detail/schistosomiasis.

[4] Carvalho OS. Moluscos Hospedeiros Intermediários de Schistosoma Mansoni do Brasil/Omar dos Santos Carvalho. Belo Horizonte: Instituto Rene Rachou /Fundação Oswaldo Cruz, 2020; p. 124.

[5] Paraense WL. *Biomphalaria straminea*, *B. tenagophila* and *B. occidentalis*: comparative anatomy, histology and susceptibility to *Schistosoma mansoni* . Mem. Inst. Oswaldo Cruz, 1986; 81: 87–97.3796282

[6] Gomes SR, Fernandez MA, Silva EF The Oswaldo Cruz Institute Mollusk Collection: improvements on diversity and infrastructure over the last years. Arq. Ciên. Mar, 2016; 49: 60–68.

[7] Varga I, Van D. Fronteiras da urbanidade sanitária: sobre o controle da malária. Rev. Saúde Pública, 2007; 16: 28–44.

[8] Souto ADSS, Thiengo SC. GBIF Coleção de Moluscos do Instituto Oswaldo Cruz. Fundação Oswaldo Cruz. Occurrence dataset. 2023; 10.15468/rptb83.

[9] Paraense WL. Estado atual da sistemática dos planorbídeos brasileiros. Arq. Mus. Nac., 1975; 55: 105–128.

[10] Simone LR. Land and Freshwater Molluscs of Brazil. São Paulo: Editora EGB, Fapesp, 2006; p. 390.

[11] Fernandez MA, Thiengo SC, Amaral R. Técnicas Malacológicas. Vigilância e Controle de Moluscos de Moluscos de Importância Médica: Diretrizes Técnicas. Editora do Ministério da Saúde, 2008; pp. 43–70.

[12] Santos SB, Ovando XMC, Lacerda EM. Planorbioidea, Planorbidae, Ancylinae. In: Damborenea C, Rogers DC, Thorp HJ (eds), Keys to Neotropical and Antarctic Fauna. Academic Press, 2020; pp. 302–307.

[13] Paraense WL. *Australorbis intermedius* sp. n. from Brazil. Rev. Bras. Biol., 1962; 22: 343–350.

[14] Paraense WL. *Biomphalaria amazonica* and B. cousini, two new species of Neotropical planorbid molluscs. Rev. Bras. Biol., 1966; 26: 115–126. 5998785

[15] Paraense WL. The Brazilian species of *Drepanotrema*. IX: *D. pileatum* sp. n. Rev. Bras. Biol., 1971; 31: 271–276. 5852741

[16] Paraense WL. *Biomphalaria oligoza* n. n. for *Tropicorbis philippianus* (Dunker) sensu Lucena. Rev. Bras. Biol., 1974; 34: 379–386.

[17] Paraense WL. *Lymnaea viatrix*: a study of topotypic specimens (Mollusca: Lymnaeidae). Rev. Bras. Biol., 1976; 36: 419–428.

[18] Paraense WL. *Biomphalaria occidentalis* sp. n. from South America (Mollusca Basommatophora Pulmonata). Mem. Inst. Oswaldo Cruz, 1981; 76: 199–211. 7348775 10.1590/s0074-02761981000200011

[19] Paraense WL. *Lymnaea rupestris* sp. n. from southern Brazil (Pulmonata: Lymnaeidae). Mem. Inst. Oswaldo Cruz, 1982; 77: 437–443.

[20] Paraense WL. *Lymnaea diaphana*: a study of topotypic specimens (Pulmonata: Lymnaeidae). Mem. Inst. Oswaldo Cruz, 1984; 79: 75–81.

[21] Paraense WL. *Biomphalaria kuhniana* (Clessin, 1883), planorbid mollusc from South America. Mem. Inst. Oswaldo Cruz, 1988; 83: 1–12.3150989

[22] Paraense WL. *Biomphalaria obstructa* (Morelet, 1849): a study of topotypic specimens (Mollusca: Pulmonata: Planorbidae). Mem. Inst. Oswaldo Cruz, 1990; 85: 391–399.

[23] Paraense WL. *Biomphalaria subprona* (Martens, 1899 (Gastropoda: Planorbidae). Mem. Inst. Oswaldo Cruz, 1996; 91: 187–190. 8736087 10.1590/s0074-02761996000200011

[24] Paraense WL. *Plesiophysa dolichomastix* sp. n. (Gastropoda: Planorbidae). Mem. Inst. Oswaldo Cruz, 2002; 97: 505–508. 12118280 10.1590/s0074-02762002000400009

[25] Paraense WL, Pointier JP. *Physa acuta* Draparnaud 1805 (Gastropoda: Physidae): a study of topotypic specimens. Mem. Inst. Oswaldo Cruz, 2003; 98: 513–517. 12937765 10.1590/s0074-02762003000400016

[26] Thiengo SC, Fernandez MA, Boaventura MF Freshwater snails and schistosomiasis mansoni in the state of Rio de Janeiro, Brazil: I- Metropolitan Mesoregion. Mem. Inst. Oswaldo Cruz, 2001; 96: 177–184. 11586447 10.1590/s0074-02762001000900028

[27] Thiengo SC, Fernandez MA, Boaventura MFF Freshwater snails and schistosomiasis mansoni in the State of Rio de Janeiro, Brazil: III – Baixada Mesoregion. Mem. Inst. Oswaldo Cruz, 2002; 97: 43–46. 10.1590/s0074-02762002000900010 12426593

[28] Thiengo SC, Fernandez AM, Boaventura MF Freshwater snails and schistosomiasis mansoni in the state of Rio de Janeiro, Brazil. II- Centro Fluminense Mesoregion. Mem. Inst. Oswaldo Cruz, 2002; 97: 621–626. 12219122 10.1590/s0074-02762002000500004

[29] Thiengo SC, Mattos AC, Boaventura MFF Freshwater snails and schistosomiasis mansoni in the State of Rio de Janeiro, Brazil: V - Norte Fluminense Mesoregion. Mem. Inst. Oswaldo Cruz, 2004; 99: 99–103. 15486644 10.1590/s0074-02762004000900018

[30] Thiengo SC, Mattos AC, Boaventura MFF Freshwater snails and schistosomiasis mansoni in the State of Rio de Janeiro, Brazil: IV - Sul Fluminense Mesoregion. Mem. Inst. Oswaldo Cruz, 2004; 99: 275–280. 15273799 10.1590/s0074-02762004000300006

[31] Thiengo SARC, Santos SB, Fernandez MA. Malacofauna límnica da área de influência do lago da usina hidrelétrica de Serra da Mesa, Goiás, Brasil: I. Estudo qualitativo. Rev. Bras. Zool., 2005; 22: 867–874.

[32] Thiengo SC, Mattos AC, Santos SB Freshwater snails and schistosomiasis mansoni in the state of Rio de Janeiro, Brazil: VI - Noroeste Fluminense Mesoregion. Mem. Inst. Oswaldo Cruz, 2006; 101: 239–245. 10.1590/s0074-02762006000900037 17308776

[33] Thiengo ST, Fernandez MA. Moluscos. In: Vigilância e controle de moluscos de importância epidemiológica: diretrizes técnicas: Programa de Vigilância e Controle da Esquistossomose. Brasília: Ministério da Saúde, Departamento de Vigilância Epidemiológica, 2008; pp. 13–36, ISBN 978-85-334-1438-9.

[34] Fiocruz/CMIOC - Coleção de Moluscos do Instituto Oswaldo Cruz. v1.48. FIOCRUZ - Oswaldo Cruz Foundation. Dataset/Occurrence. http://ipt.fiocruz.br/ipt/resource?r=fiocruz_cmioc&amp;v=1.48.

[35] Vectors of Human Disease Series. *GigaByte*. 2022; 10.46471/GIGABYTE_SERIES_0002.

